# Plant catalase in silico characterization and phylogenetic analysis with structural modeling

**DOI:** 10.1186/s43141-022-00404-6

**Published:** 2022-08-19

**Authors:** Takio Nene, Meera Yadav, Hardeo Singh Yadav

**Affiliations:** Department of Chemistry, North Eastern Regional Institute of Science and Technology, Itanagar, India

**Keywords:** Catalase, Phylogenetic, Homology modeling, Thermostable, In silico

## Abstract

**Background:**

Catalase (EC 1.11.1.6) is a heme-containing tetrameric enzyme that plays a critical role in signaling and hydrogen peroxide metabolism. It was the first enzyme to be crystallized and isolated. Catalase is a well-known industrial enzyme used in diagnostic and analytical methods in the form of biomarkers and biosensors, as well as in the textile, paper, food, and pharmaceutical industries. In silico *analysis* of CAT genes and proteins has gained increased interest, emphasizing the development of biomarkers and drug designs. The present work aims to understand the catalase evolutionary relationship of plant species and analyze its physicochemical characteristics, homology, phylogenetic tree construction, secondary structure prediction, and 3D modeling of protein sequences and its validation using a variety of conventional computational methods to assist researchers in better understanding the structure of proteins.

**Results:**

Around 65 plant catalase sequences were computationally evaluated and subjected to bioinformatics assessment for physicochemical characterization, multiple sequence alignment, phylogenetic construction, motif and domain identification, and secondary and tertiary structure prediction. The phylogenetic tree revealed six unique clusters where diversity of plant catalases was found to be the largest for *Oryza sativa*. The thermostability and hydrophilic nature of these proteins were primarily observed, as evidenced by a relatively high aliphatic index and negative GRAVY value. The distribution of 5 sequence motifs was uniformly distributed with a width length of 50 with the best possible amino residue sequences that resemble the plant catalase PLN02609 superfamily. Using SOPMA, the predicted secondary structure of the protein sequences revealed the predominance of the random coil. The predicted 3D CAT model from *Arabidopsis thaliana* was a homotetramer, thermostable protein with 59-KDa weight, and its structural validation was confirmed by PROCHECK, ERRAT, Verify3D, and Ramachandran plot. The functional relationships of our query sequence revealed the glutathione reductase as the closest interacting protein of query protein.

**Conclusions:**

This theoretical plant catalases in silico analysis provide insight into its physiochemical characteristics and functional and structural understanding and its evolutionary behavior and exploring protein structure-function relationships when crystal structures are unavailable.

## Background

Catalases (EC 1.11.1.6) are iron porphyrin oxidoreductase enzymes that scavenge hydrogen peroxide into water and oxygen [1, 2]. They are heme-containing tetrameric enzymes found in subcellular organelles (peroxisomes), the primary source of H_2_O_2_ production during oxidative stress conditions via photorespiratory oxidation, beta oxidation of fatty acids, and purine catabolism [3]. CAT plays a crucial role due to pathological events connected to their dysfunction, such as increased vulnerability to apoptosis, tumor stimulation, regulated aging, and inflammation. It also aids in defensive mechanisms and protects the cell from oxidative damage. Another significant property of catalase is its strong catalytic activity, using H_2_O_2_ as a substrate to oxidize phenols, insecticides, herbicides, polyaromatic hydrocarbons, and synthetic textile dyes [4]. Catalase was the first enzyme to crystallize and isolate. They are found in various plant species such as tobacco, *Arabidopsis thaliana*, pepper, mustard, saffron, maize, castor bean, sunflower, cotton, wheat, and spinach [5–11]. The role of catalase in aging, senescence, and plant defense has been of significant importance. In light of the different applications of catalase mentioned above, the current work is being conducted for in silico analysis from plant sources. Computational investigation of the plant catalase amino sequence revealed the conserved secondary structure in sequences that play a crucial role in evolution. Primary research on catalases was conducted to examine their characteristics and key biological functions. Analyses of the phylogeny of the catalase gene has indicated the existence of three primary clades that separated themselves early in the evolution of this gene family by at least two gene duplication events [12]. A phylogenetic approach could help us account for the intrinsic divergence in enzyme dynamics induced by the natural evolution of sequence variation across time [13]. As genomics advances, computational tools are becoming increasingly crucial in helping to find and describe possible gene families for various industrial uses. This helps untangle the sequence-structure-functional relationship between enzyme protein sequences [14]. The analysis of genes and proteins in silico has gained increased interest, emphasizing the development of biomarkers, drug design, and the development of a very effective microbiological agent suitable for a wide range of industries. The present work aims to understand the catalase evolutionary relationship of plant species and analyze its physicochemical characteristics, homology, phylogenetic tree construction, secondary structure prediction, and 3D modeling of protein sequences and its validation using a variety of conventional computational methods to assist researchers in better understanding the structure of proteins.

## Methods

### Protein sequence recovery

In FASTA format for various computational analyses, sixty-five full-length catalase protein sequences from various plant sources were retrieved from the NCBI (National Center for Biotechnology Information) database. The number of protein sequences with accession numbers and source organisms is given in Table 1.

**Table 1:** Selected protein sequences of catalases from different plant sources

Sl. no.	Source organisms	Accession number of protein sequence retrieved	Number of sequences
1	*Vigna radiata*	NP 001304079, BAA02755, ADZ45556, ADZ45555	4
2	*Populus deltoides*	CAI43948	1
3	*Ziziphus jujuba*	AET97564	1
4	*Prunus persica*	CAD42908, CAB56850, CAD42909	3
5	*Phyllanthus emblica*	ATO98311	1
6	*Nicotiana plumbaginifolia*	CAA85426, CAA85424	2
7	*Bruguiera gymnorhiza*	ADC95629	1
8	*Arabidopsis thaliana*	CAB80226, CAA17773, CAA45564	3
9	*Raphanus sativus*	AAF71742	1
10	*Brassica juncea*	AAD17934, AAD17936, AAD17935, AAD17933	4
11	*Arabis alpina*	KFK30147	1
12	*Musa acuminata*	SIW58963	1
13	*Solanum tuberosum*	AAR14052, AAA80650, CAA85470	3
14	*Vitis vinifera*	NP 001268098, AAL83720	2
15	*Saccharum*	AIU99487, AIU99488, AIM43584, AIU99482	4
16	*Saccharum spontaneum*	AIU99481, AIU99480, AIU99485, AIU99486	4
17	*Saccharum arundinaceum*	AIU99484	1
18	*Oryza sativa*	AKO90140, BAA34204, BAA05494, BAA34205, BAA34714, BAA06232, CAA43814, BAA81677, BAA81672, BAA81671, BAA81670	11
19	*Triticum aestivum*	ADF83496, BAA13068	2
20	*Festuca arundinacea*	CAG23920	1
21	*Capsicum annuum*	NP 001311603, BAF91369, AAF34718	3
22	*Solanum melongena*	CAA50644	1
23	*Solanum lycopersicum*	AAA34145	1
24	*Oryza meridionalis*	BAA81679, BAA81678	2
25	*Oryza rufipogon*	BAA81676, BAA81675, BAA81674, BAA81673	4
26	*Oryza glaberrima*	BAA81682, BAA81681	2
27	*Oryza barthii*	BAA81680	1

### ProtParam tool for primary sequence analysis

The ExPasy ProtParam tool was used to compute the physiochemical parameters of the selected catalases. ProtParam calculates a variety of physicochemical properties that can be derived from the sequence of a protein. The molecular weight, theoretical pI, amino acid composition, atomic composition, extinction coefficient, estimated half-life, instability index, aliphatic index, and grand average of hydropathicity (GRAVY) are all parameters computed by ProtParam [15] (http://web.expasy.org/protparam/).

### Multiple Sequence Alignment (MSA)

The multiple sequence alignment of protein profiles was developed using MEGA 6.1 software to verify the accuracy of the alignment. The ClustalW program was used to perform multiple alignments of sequences.

### Amino acid composition

MEGA 11 examined the catalase-encoding amino acid composition where all species’ individual amino acid frequencies were retrieved (https://www.megasoftware.net/).

### Phylogenetic tree construction

To better understand the evolutionary relationships between plant species, catalase phylogenetic trees were constructed with MEGA6 software, and the visualization of phylogenetic tree patterns was performed using the neighbor-joining (NJ) method or UPGMA [16].

### Motifs search and domain discovery

The analysis of motifs was done using the MEME tool (http://meme.sdsc.edu/meme/meme.html), which was also used to search their protein family using the NCBI conserved domain database (CDD) (https://www.ncbi.nlm.nih.gov/Structure/cdd/wrpsb.cgi). The biological activities of conserved protein motif data collected by MEME were analyzed using BLAST, and domains were assessed using InterProScan by offering the most significant possible match of sequences based on their highest similarity score [17].

### Prediction of secondary structure

Secondary structures have a direct impact on how proteins fold and deform. This is how various amino acid sequences of plant catalase form helixes, sheets, and turns in the molecule. SOPMA (self-optimized prediction method with alignment) was used to predict the secondary structure of different plant catalases [18]. It is a self-optimized homologous tool based on Levin and his colleagues [19].

### Comparative 3D modeling

A query protein sequence from each cluster group generated from a phylogenetic tree of plant catalase was analyzed, and comparative homology modeling was performed using the SWISS-MODEL (http://swissmodel.expasy.org) [20], based on automated comparative 3D modeling of protein structures.

### Model evaluation

The most crucial step in homology modeling is model evaluation, which demonstrates that the modeled protein is of acceptable quality. Here, the predicted CAT model was evaluated and verified by the ERRAT value [21], Verify3D score [22], and PROCHECK [23] programs available from the SAVES server (http://nihserver.mbi.ucla.edu/SAVES). The quality of the predicted model was evaluated by Ramachandran plot assessment.

### Protein-protein interaction

STRING v10.0 (http://string-db.org/) server was used to determine the catalase interaction of *Arabidopsis thaliana* with other closely related proteins. The query sequence was *Arabidopsis thaliana* with accession number CAA45564.1, and a functional protein association network was created [24].

## Results

### Retrieval of sequences

The protein sequences of many enzymes like peroxidases [25–27], pectinases, proteases [28], lipases [29], phytases, polyphenol oxidases [15], and cellulases [29] have been assessed and analyzed using bioinformatics tools. The current study used various bioinformatic tools to analyze the protein sequences of industrially important enzyme catalases from various plant sources. Around 150 catalase protein sequences from various plant sources were initially retrieved from NCBI using the BLAST method. From there, sequences with more than 70% similarity were selected where only 65 sequences were computationally evaluated based on full-length protein sequences (see Table 1). The diversity of plant sources for catalases was observed and found the largest for *Oryza sativa*, with 11 accession numbers forming the main group. *Oryza sativa* consists of four catalase genes OsCATA, OsCATB, OsCATC, and OsCATD [30], with functional variations under various abiotic stress conditions. Multiple accessions of the same catalase source help us gain insight into the structural and functional diversity of enzymatic proteins.

### Physicochemical characterization

ProtParam was used to elucidate several physiochemical properties of the sequences. The amino acid residue variability in the 65 catalase protein sequences studied ranged from 90 to 533. The molecular weights varied between 10,322.46 and 61,366.87 daltons, while the pI values varied between 4.53 and 7.95. Most catalases had pI ranging from 5 to 7, while AAF34718 of *Capsicum annuum* has the pI value of 7.11, and the *Oryza* family placed in group F of the phylogenetic tree showed pI ranging from 4 to 5. Other physicochemical characteristics such as instability index, aliphatic index, and hydropathicity (GRAVY) were also variable for these CAT proteins. The aliphatic index measures the relative volume filled by the aliphatic side chain of amino acids such as alanine, valine, leucine, and isoleucine and provides information on the thermostability of globular proteins. It may be seen positively in increasing the thermostability of globular proteins. The following formula is used to determine the aliphatic index [31].

$$\mathrm{Aliphatic}\ \mathrm{index}=\mathrm{X}\ \left(\mathrm{Ala}\right)+\mathrm{a}\times \mathrm{X}\ \left(\mathrm{Val}\right)+\mathrm{b}\times \left(\mathrm{X}\ \left(\mathrm{Ile}\right)+\mathrm{X}\ \left(\mathrm{Leu}\right)\right)$$

The coefficients a and b are the relative volume of valine side chain (a = 2.9) and of Leu/Ile side chains (b = 3.9) to the side chain of alanine.

Plant catalases are assumed to be thermostable based on the data shown in Table 2. The instability index represents the in vivo half-life of a protein, and a number greater than 40 suggests a half-life of less than 5 h, while a value less than 40 indicates a half-life of more than 16 h. It also estimates the stability of the protein molecule [32, 33]. Most plant catalases have an instability index of less than 40, except a few that belong to the *Oryza*, *Capsicum annuum*, and *Brassica juncea* families. The hydrophobicity value of a peptide is represented by the grand average hydropathicity index (GRAVY), which is calculated as the sum of the hydropathy values of all amino acids divided by the sequence length, revealing that the negative value of the obtained plant proteins is hydrophilic.

**Table 2:** Physiochemical characterization of protein sequences of plant catalases as revealed by ProtParam

S. no.	Accession number	Source organisms	No. of amino acids	Molecular weight	Theoretical pI	Total number of negatively charged residues (Asp + Glu)	Total number of positively charged residues (Arg + Lys)	Instability index	Aliphatic index	Grand average of hydropathicity (GRAVY)
1	NP001304079	*Vigna radiata*	514	58955.50	6.69	63	59	38.76	73.81	−0.488
2	BAA02755	*Vigna radiata* var. *radiata*	525	60026.27	6.82	64	61	40.12	74.70	−0.462
3	ADZ45556	*Vigna radiata*	514	59000.14	6.58	64	59	38.33	73.30	−0.493
4	ADZ45555	*Vigna radiata*	515	58990.10	6.58	64	59	38.70	73.30	−0.492
5	CAI43948	*Populus deltoides*	519	59759.30	6.30	65	57	38.87	71.97	−0.479
6	AET97564	*Ziziphus jujuba*	492	57012.28	6.78	61	58	36.67	71.34	−0.586
7	CAD42908	*Prunus persica*	516	59502.85	6.67	65	61	41.93	70.66	−0.563
8	ATO98311	*Phyllanthus emblica*	206	23309.00	7.95	19	20	37.11	72.43	−0.176
9	CAA85426	*Nicotiana plumbaginifolia*	527	60803.50	6.68	62	58	38.75	72.73	−0.436
10	ADC95629	*Bruguiera gymnorhiza*	522	60289.99	6.99	62	60	39.96	72.30	−0.521
11	CAB80226	*Arabidopsis thaliana*	527	60803.50	6.68	62	58	38.75	72.73	−0.436
12	CAA17773	*Arabidopsis thaliana*	522	59978.31	6.56	64	59	39.65	72.15	−0.508
13	CAA45564	*Arabidopsis thaliana*	522	59932.31	6.67	63	59	39.65	74.02	−0.484
14	AAF71742	*Raphanus sativus*	518	59445.81	6.67	63	59	40.80	70.04	−0.521
15	AAD17934	*Brassica juncea*	492	56828.17	6.63	62	58	41.11	70.14	−0.571
16	AAD17936	*Brassica juncea*	492	56946.31	6.63	62	58	39.93	70.14	−0.569
17	KFK30147	*Arabis alpina*	515	59241.56	7.15	63	62	42.87	71.59	−0.529
18	AAD17935	*Brassica juncea*	492	56915.30	6.90	61	59	41.58	69.53	−0.581
19	AAD17933	*Brassica juncea*	496	57411.82	6.75	62	59	41.81	68.97	−0.574
20	SIW58963	*Musa acuminata*	289	32993.15	5.60	35	25	33.43	75.57	−0.248
21	AAR14052	*Solanum tuberosum*	509	58871.71	6.76	62	59	36.25	72.81	−0.496
22	CAA85424	*Nicotiana plumbaginifolia*	527	60359.75	6.73	61	58	42.60	72.54	−0.431
23	CAB56850	*Prunus persica*	519	60050.61	6.44	66	60	38.94	72.33	−0.525
24	CAD42909	*Prunus persica*	516	59586.90	6.83	66	63	43.98	70.68	−0.582
25	NP 001268098	*Vitis vinifera*	515	59395.13	6.60	64	60	36.37	71.53	−0.487
26	AAL83720	*Vitis vinifera*	516	59439.22	6.61	64	60	36.52	72.54	−0.462
27	AIU99487	*Saccharum* hybrid cultivar ROC22	529	61053.51	7.23	61	60	35.78	70.96	−0.459
28	AIU99488	*Saccharum* hybrid cultivar ROC22	529	61049.50	7.23	61	60	34.80	71.70	−0.454
29	AIU99481	*Saccharum spontaneum*	522	60096.68	6.65	62	57	34.06	70.06	−0.475
30	AIU99480	*Saccharum spontaneum*	522	60137.74	6.76	62	58	33.13	71.36	−0.489
31	AIU99484	*Saccharum arundinaceum*	524	60310.91	6.65	63	58	33.58	71.83	−0.462
32	AIU99485	*Saccharum spontaneum*	522	60074.72	6.76	61	57	33.26	69.67	−0.476
33	AIM43584	*Saccharum* hybrid cultivar Yacheng05-179	533	61366.87	6.79	63	59	33.08	71.16	−0.464
34	AIU99486	*Saccharum spontaneum*	522	60105.77	6.65	62	57	33.82	70.79	−0.461
35	AIU99482	*Saccharum* hybrid cultivar ROC22	529	61091.49	6.89	63	60	33.32	72.25	−0.461
36	AKO90140	*Oryza sativa*	514	59001.53	6.69	63	59	28.94	72.67	−0.476
37	BAA34204	*Oryza sativa* Japonica Group	492	56575.00	6.49	62	56	31.71	70.73	−0.521
38	BAA05494	*Oryza sativa* Japonica Group	492	56518.89	6.47	62	56	29.80	70.35	−0.519
39	ADF83496	*Triticum aestivum*	519	59739.15	6.35	66	59	36.54	70.08	−0.520
40	BAA13068	*Triticum aestivum*	519	59662.62	6.44	65	59	37.70	70.27	−0.522
41	CAG23920	*Festuca arundinacea*	521	59642.71	6.13	68	58	35.71	68.71	−0.551
42	BAA34205	*Oryza sativa* Japonica Group	492	56806.00	6.93	60	58	34.56	70.73	−0.583
43	BAA34714	*Oryza sativa*	514	58477.81	7.44	59	59	38.40	65.66	−0.570
44	NP001311603	*Capsicum annuum*	516	59027.52	6.89	61	59	41.91	71.24	−0.456
45	BAF91369	*Capsicum annuum*	517	59149.21	6.89	61	59	42.05	71.30	−0.443
46	AAF34718	*Capsicum annuum*	517	59102.64	7.11	61	60	42.02	71.12	−0.439
47	CAA50644	*Solanum melongena*	519	59612.46	6.49	65	60	38.12	72.52	−0.462
48	AAA80650	*Solanum tuberosum*	519	59490.24	6.46	65	60	40.91	67.65	−0.536
49	AAA34145	*Solanum lycopersicum*	522	59953.61	6.46	65	60	39.89	70.25	−0.489
50	CAA85470	*Solanum tuberosum*	525	60195.17	6.58	65	61	38.90	70.23	−0.501
51	BAA06232	*Oryza sativa*	513	58948.73	6.78	65	62	40.97	68.42	−0.521
52	CAA43814	*Oryza sativa* Indica Group	524	60255.59	6.77	66	63	39.88	68.66	−0.496
53	BAA81679	*Oryza meridionalis*	119	13672.55	4.55	21	11	39.18	75.55	−0.396
54	BAA81678	*Oryza meridionalis*	119	13672.55	4.55	21	11	39.18	75.55	−0.396
55	BAA81677	*Oryza sativa* f. *spontanea*	123	13890.66	4.53	20	10	40.11	68.37	−0.396
56	BAA81676	*Oryza rufipogon*	116	13488.20	4.70	19	11	38.85	66.55	−0.474
57	BAA81675	*Oryza rufipogon*	116	13488.20	4.70	19	11	38.85	66.55	−0.474
58	BAA81674	*Oryza rufipogon*	121	13958.73	4.72	20	12	38.55	67.02	−0.469
59	BAA81673	*Oryza rufipogon*	121	13958.73	4.72	20	12	38.55	67.02	−0.469
60	BAA81672	*Oryza sativa*	120	13504.30	4.84	20	13	39.12	71.67	−0.436
61	BAA81671	*Oryza sativa* Indica Group	131	14831.73	4.86	21	14	37.90	72.37	−0.401
62	BAA81670	*Oryza sativa* Japonica Group	90	10322.46	4.57	19	10	39.95	63.89	−0.700
63	BAA81682	*Oryza glaberrima*	119	13476.86	4.86	20	13	43.60	69.83	−0.492
64	BAA81681	*Oryza glaberrima*	117	13291.63	4.74	20	12	44.90	71.03	−0.463
65	BAA81680	*Oryza barthii*	115	13113.42	4.95	19	12	42.53	71.39	−0.492

### Assessment of phylogenetic tree and MSA

**Fig. 1:**
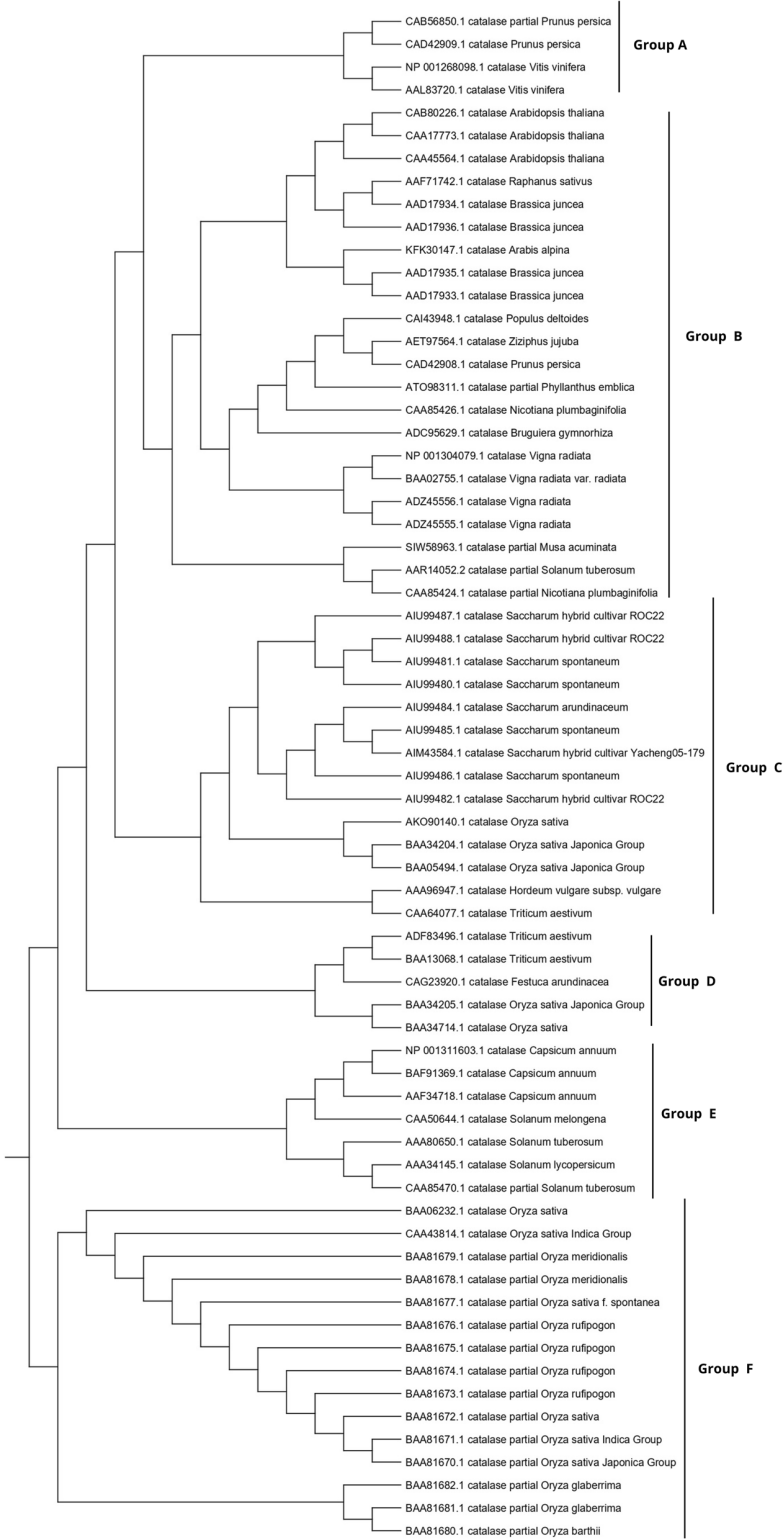
Construction of phylogenetic tree of protein sequences of plant catalases using NJ method. The unique clusters **A, B, C, D, E**, and **F** are highlighted, consisting of 4, 22, 12, 5, 7, and 15 members, respectively

**Fig. 2:**
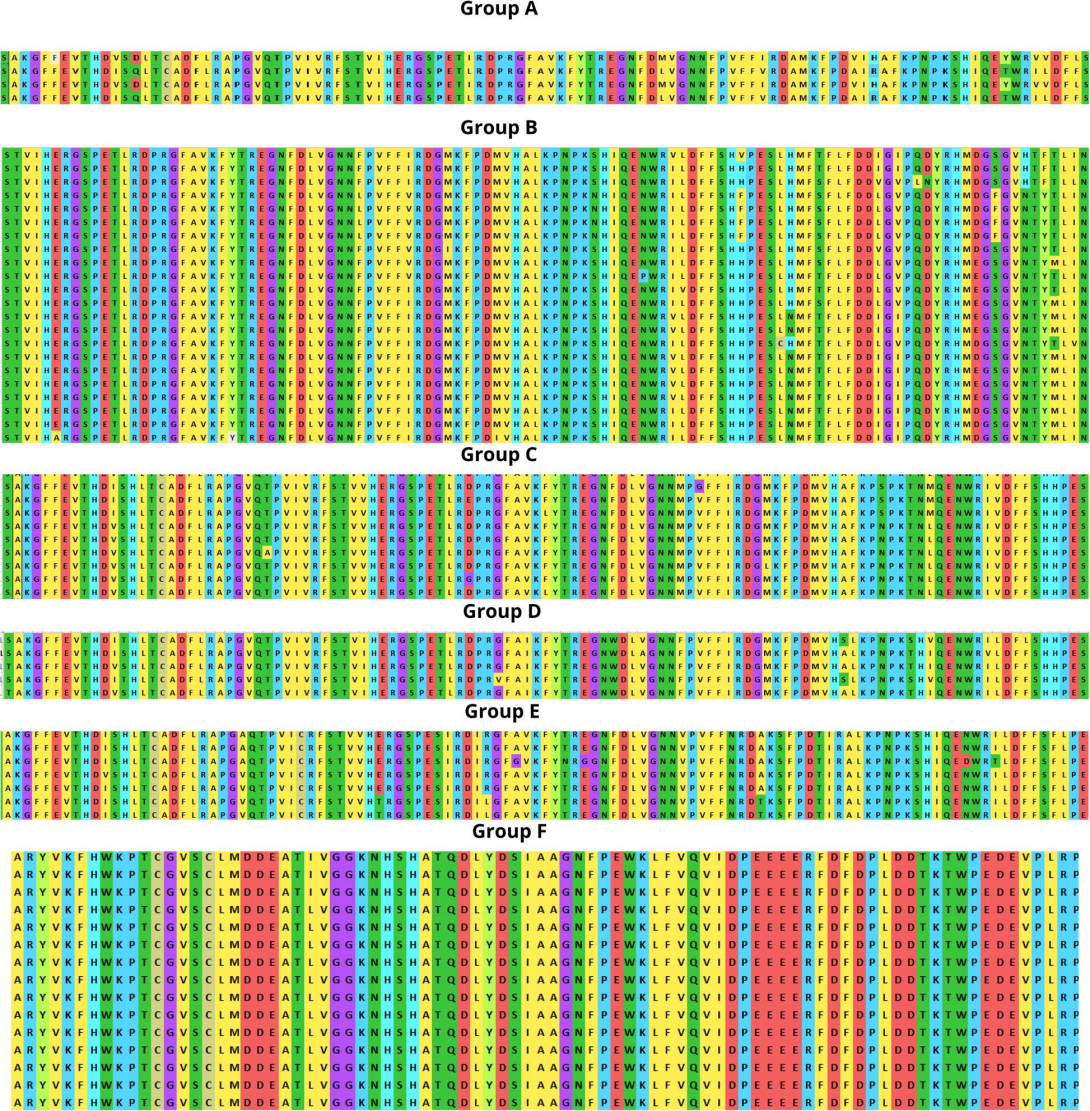
Multiple sequence alignment of distinct clusters **A, B, C, D, E**, and **F** of plant catalases

The phylogenetic tree revealed six unique clusters labeled A, B, C, D, E, and F, each of which had 4, 22, 12, 5, 7, and 15 protein sequences are shown in Fig. 1. Multiple accessions belonging to the same genus were grouped, suggesting similarity at the sequence level, except for the *Oryza sativa* protein sequence was distributed in both groups D and F. The phylogenetic analysis provides a depth understanding of how species evolve due to genetic alterations. Scientists can use phylogenetics to examine the path that connects a modern plant CAT organism to its ancestral origin and anticipate future genetic divergence. It can also be helpful in comparative genomics, which analyzes the relationship between genomes of different species by gene prediction or discovery, locating specific genetic regions along a genome [34–36]. Before building the phylogenetic tree, the alignment of multiple sequences is shown in Fig. 2, revealing the degree of homology between the sequences from different plant sources. This information could be used to synthesize a specific catalase probe or primer that would serve as a marker to remove putative genes from sequenced plant strains. The advancement in the comparative genomic study of proteins provides a detailed understanding of functional genes within and between plant species, providing clear evidence for evolution research and gene function hypotheses of plant catalase [37].

### Motifs and domain identification

The structure and functional complexity of enzymes can be predicted and assessed using attributes such as sequence and function order features, domains, and motifs. Sequence motifs identified by protein sequence analysis can be used as signature sequences for targeted enzymes to determine their putative functions [38–40]. The distribution of 5 sequence motifs among 65 plant catalases was analyzed, uniformly distributed with a width length of 50 with the best possible amino residue sequences, as shown in Table 3. When these motifs were subjected to BLAST, they resembled the plant catalase superfamily PLN02609.

**Table 3:** The five motifs with best match possible amino acid sequences with their respective domain

Motifs	Width	Best possible amino acids	Conserved domain
1	50	KFHWKPTCGVKCLMEDEAITVGGTNHSHATQDLYDSIAAGNYPEWKLFIQ	Plant catalase PLN02609 superfamily
2	50	APGVQTPVIVRFSTVIHERGSPETLRDPRGFAVKFYTREGNFDLVGNNMP	Plant catalase PLN02609 superfamily
3	50	DFDPLDVTKTWPEDILPLQPVGRMVLNKNIDNFFAENEQLAFCPAIIVPG	Plant catalase PLN02609 superfamily
4	50	KPNPKSHIQENWRILDFFSHHPESLHMFTFLFDDVGIPQDYRHMEGSGVN	Plant catalase PLN02609 superfamily
5	50	IYYSDDKMLQTRIFSYADTQRHRLGPNYLQLPVNAPKCAHHNNHHEGFMN	Plant catalase PLN02609 superfamily

### Amino acid composition

MEGA 11 was used to compute the composition of the amino acid sequences individually. The average amino acid composition was highest for proline at 7.38%, followed by aspartate (7.12%) given in Table 4, suggesting significant conformational rigidity of the secondary structure of the protein due to the distinctive cyclic structure of the proline side chain [41].

**Table 4:** Amino acid composition (%) of CAT protein from different plant sources

Accession number	Ala	Cys	Asp	Glu	Phe	Gly	His	Ile	Lys	Leu	Met	Asn	Pro	Gln	Arg	Ser	Thr	Val	Trp	Tyr	Total
CAB56850.1	5.91	1.18	7.09	5.67	6.86	4.73	5.44	4.96	5.44	6.86	1.42	4.96	7.33	3.31	6.86	5.91	4.02	6.38	1.89	3.78	423
NP001311603.1	6.1	1.83	6.71	5.49	6.1	5.28	4.07	5.08	5.08	6.71	1.83	5.28	7.32	2.64	6.91	5.89	5.28	6.91	1.42	4.07	492
AIU99487.1	5.49	1.83	6.91	5.08	6.71	5.69	5.49	4.88	4.88	6.91	2.44	5.89	6.91	3.05	6.5	4.88	5.28	6.5	1.83	2.85	492
AIU99484.1	5.49	1.83	6.91	5.28	6.5	5.49	5.49	5.08	4.88	7.11	2.24	5.89	7.11	3.05	6.5	4.47	5.28	6.71	1.83	2.85	492
ADF83496.1	5.49	0.81	7.32	5.49	6.1	5.49	4.47	4.67	4.67	7.11	2.24	5.49	7.52	2.24	7.11	6.5	5.28	6.1	2.03	3.86	492
CAB80226.1	5.69	1.22	6.71	5.89	6.71	5.28	4.47	6.1	4.88	6.3	2.03	6.1	7.52	3.05	6.91	5.69	4.47	5.69	1.63	3.66	492
BAA34205.1	5.28	0.81	7.72	4.47	6.3	5.49	4.88	5.08	4.47	7.32	1.83	5.49	7.72	2.44	7.32	6.91	4.88	5.89	2.24	3.46	492
CAI43948.1	6.1	1.63	6.5	5.69	6.5	5.69	4.67	5.08	4.88	6.91	1.63	5.28	7.52	2.85	6.71	6.1	4.27	6.3	2.03	3.66	492
CAA45564.1	5.89	1.22	6.71	5.69	6.71	5.28	4.47	6.3	4.88	6.71	1.83	6.1	7.32	3.05	6.91	5.69	4.47	5.49	1.63	3.66	492
NP001304079.1	6.5	0.61	6.71	5.69	7.32	5.28	4.88	5.28	4.88	6.71	1.63	6.3	7.11	2.64	6.91	5.69	4.07	6.71	2.03	3.05	492
NP001268098.1	5.69	1.02	6.71	5.89	7.32	5.28	4.07	4.47	5.28	6.3	1.83	5.69	7.32	2.85	6.71	5.49	4.88	7.32	1.42	4.47	492
SIW58963.1	3.49	0.78	7.75	5.43	8.91	6.59	4.65	5.43	4.26	7.36	2.33	5.81	7.36	2.71	5.04	5.04	5.43	6.98	1.55	3.1	258
AAR14052.2	5.47	1.26	6.95	5.68	6.53	5.47	4.42	5.89	4.63	6.74	1.68	5.47	6.53	3.37	7.16	6.11	4.84	6.32	1.68	3.79	475
AKO90140.1	5.69	1.83	7.52	5.08	6.1	5.69	4.88	4.88	4.88	7.52	2.44	5.69	7.11	2.64	6.71	5.28	5.08	6.3	1.83	2.85	492
AIU99488.1	5.49	1.83	6.71	5.28	6.71	5.69	5.49	5.08	4.88	6.91	2.24	5.89	6.91	3.05	6.5	4.88	5.28	6.5	1.83	2.85	492
AIU99486.1	5.28	1.83	6.91	5.28	6.71	5.69	5.49	5.08	4.88	6.91	2.24	5.89	6.91	2.85	6.5	4.67	5.28	6.91	1.83	2.85	492
AIU99485.1	5.49	1.83	6.71	5.28	6.71	5.69	5.49	5.08	4.88	6.71	2.24	5.89	7.11	3.05	6.5	4.67	5.28	6.71	1.83	2.85	492
AIU99482.1	5.49	1.83	7.11	5.28	6.5	5.49	5.49	5.08	4.88	6.91	2.24	5.69	6.71	3.25	6.5	4.88	5.28	6.71	1.83	2.85	492
AIU99481.1	5.69	1.83	6.91	5.28	6.71	5.49	5.49	5.08	4.88	6.91	2.24	5.89	6.71	3.05	6.5	5.08	5.08	6.5	1.83	2.85	492
AIU99480.1	5.49	1.63	6.91	5.28	6.5	5.49	5.49	5.08	4.88	7.32	2.03	5.89	6.91	3.05	6.71	4.88	5.28	6.5	1.83	2.85	492
AIM43584.1	5.49	1.83	6.91	5.28	6.71	5.49	5.49	4.88	4.88	6.91	2.24	5.89	6.91	3.05	6.5	4.67	5.49	6.71	1.83	2.85	492
KFK30147.1	5.89	1.22	6.5	6.1	6.71	5.28	4.47	5.69	4.67	6.3	2.03	6.1	7.72	2.64	7.32	5.89	4.07	6.1	1.63	3.66	492
AET97564.1	5.89	1.22	6.71	5.69	6.5	5.28	4.88	5.69	4.88	6.71	1.42	5.89	7.52	3.05	6.91	5.89	4.07	5.89	2.03	3.86	492
ADZ45556.1	6.5	0.81	6.71	5.69	7.11	5.28	4.88	5.28	4.88	6.71	1.63	6.1	7.32	2.64	6.91	5.69	4.27	6.5	2.03	3.05	492
ADZ45555.1	6.5	0.81	6.71	5.69	7.11	5.28	4.88	5.28	4.88	6.71	1.63	6.1	7.11	2.64	6.91	5.89	4.27	6.5	2.03	3.05	492
AAA80650.1	5.89	2.03	7.11	5.69	5.89	5.89	3.86	4.88	5.08	6.3	1.63	5.49	7.32	2.64	6.91	5.89	5.28	6.5	1.42	4.27	492
AAA34145.1	5.89	2.03	6.91	5.89	5.89	5.49	3.86	5.08	5.08	6.5	1.63	5.49	7.52	2.64	6.91	5.69	5.28	6.5	1.42	4.27	492
AAF71742.1	5.49	1.22	6.5	6.1	6.71	5.49	4.47	5.49	4.88	6.5	2.03	6.1	7.72	2.64	6.91	6.1	4.27	6.1	1.63	3.66	492
AAF34718.1	6.5	1.83	6.5	5.69	6.1	5.28	4.07	5.08	5.08	6.5	1.83	5.28	7.32	2.64	7.11	5.89	4.88	6.91	1.42	4.07	492
ADC95629.1	5.89	1.63	6.5	5.49	6.5	5.49	4.88	5.49	4.47	6.91	1.22	6.1	7.52	2.85	7.11	5.49	4.27	6.5	2.03	3.66	492
AAL83720.1	5.69	1.02	6.71	5.89	7.32	5.28	4.07	4.47	5.28	6.3	1.83	5.69	7.32	2.85	6.71	5.49	4.88	7.32	1.42	4.47	492
AAD17936.1	5.49	1.22	6.5	6.1	6.91	5.49	4.47	5.89	4.67	6.3	2.03	6.3	7.72	2.64	7.11	5.69	4.27	5.89	1.63	3.66	492
AAD17935.1	5.49	1.22	6.1	6.3	6.71	5.28	4.47	5.08	4.88	6.5	2.03	6.3	7.72	2.64	7.11	5.89	4.47	6.5	1.63	3.66	492
AAD17934.1	5.69	1.22	6.5	6.1	6.71	5.49	4.47	5.69	4.88	6.3	2.03	6.3	7.72	2.64	6.91	5.69	4.27	6.1	1.63	3.66	492
AAD17933.1	5.44	1.21	6.25	6.25	7.06	5.24	4.44	5.04	4.84	6.45	2.02	6.25	7.66	2.62	7.06	6.05	4.44	6.45	1.61	3.63	496
BAA34204.1	5.69	1.83	7.32	5.28	6.5	5.69	5.08	4.88	4.67	7.11	2.44	5.69	7.11	2.85	6.71	5.28	5.08	6.3	1.83	2.64	492
BAA05494.1	5.89	1.83	7.52	5.08	6.5	5.89	4.88	4.88	4.67	7.11	2.44	5.69	6.91	2.85	6.71	5.28	5.08	6.1	1.83	2.85	492
BAA02755.1	6.5	0.61	6.71	5.69	7.32	5.28	4.88	5.28	4.88	6.71	1.63	6.3	7.11	2.64	6.91	5.69	4.07	6.71	2.03	3.05	492
BAF91369.1	6.1	1.83	6.71	5.49	6.1	5.28	4.07	5.08	5.08	6.71	1.83	5.28	7.32	2.64	6.91	5.89	5.28	6.91	1.42	4.07	492
CAA85470.1	6.11	1.83	6.92	5.91	5.91	5.5	3.87	5.09	5.09	6.52	1.43	5.3	7.33	2.65	6.92	5.91	5.5	6.52	1.43	4.28	491
BAA06232.1	6.31	1.63	7.54	5.5	6.92	5.5	4.48	3.87	4.28	6.11	1.63	4.89	8.35	2.44	8.15	4.07	5.5	7.74	1.63	3.46	491
CAA17773.1	5.69	1.22	6.71	5.89	6.71	5.28	4.47	6.1	4.88	6.3	2.03	6.1	7.52	3.05	6.91	5.69	4.47	5.69	1.63	3.66	492
CAG23920.1	5.69	0.81	7.93	5.08	6.1	5.89	4.47	4.88	4.27	7.11	2.03	5.49	7.52	2.44	7.32	5.89	5.49	5.89	2.03	3.66	492
CAA50644.1	6.1	1.83	6.71	5.89	6.1	5.28	4.07	5.08	5.08	6.71	1.83	5.28	7.32	2.64	7.11	5.49	4.88	7.11	1.42	4.07	492
CAA85426.1	5.69	1.83	6.71	5.69	6.71	5.28	4.88	4.88	5.08	6.71	2.03	5.89	7.52	2.85	6.71	5.28	4.27	6.5	2.24	3.25	492
CAA85424.1	5.15	1.44	7.01	5.36	6.8	5.36	4.12	4.74	4.74	6.6	1.86	5.57	7.22	3.09	7.01	6.8	5.36	6.6	1.44	3.71	485
CAA43814.1	6.52	1.63	7.54	5.5	6.92	5.5	4.48	3.87	4.28	5.91	1.63	4.89	7.94	2.65	8.15	4.07	5.5	7.94	1.63	3.46	491
CAD42909.1	6.1	1.02	7.11	5.69	6.3	4.88	5.08	4.67	5.08	7.11	1.22	4.88	7.72	2.85	7.32	6.1	4.27	6.71	2.03	3.86	492
CAD42908.1	5.28	1.22	6.91	5.69	6.5	5.28	4.67	4.88	5.08	7.11	1.42	5.49	7.72	3.05	6.91	6.3	4.07	6.71	2.03	3.66	492
BAA13068.1	5.49	0.81	7.32	5.49	6.1	5.49	4.47	4.67	4.67	7.11	2.24	5.49	7.52	2.24	7.11	6.5	5.28	6.1	2.03	3.86	492
BAA34714.1	5.69	1.42	7.32	4.47	5.89	6.1	4.47	4.27	4.27	6.5	1.83	5.49	7.72	2.24	7.52	7.72	5.89	5.89	2.44	2.85	492
ATO98311.1	5.29	1.18	6.47	3.53	10	7.65	6.47	3.53	5.29	5.29	2.35	4.12	5.88	2.35	5.88	5.88	5.88	9.41	1.18	2.35	170
BAA81682.1	5.81	2.33	11.63	9.3	5.81	5.81	3.49	3.49	6.98	5.81	1.16	2.33	8.14	2.33	3.49	3.49	5.81	6.98	3.49	2.33	86
BAA81681.1	5.95	2.38	11.9	9.52	5.95	4.76	3.57	3.57	5.95	5.95	1.19	2.38	8.33	2.38	3.57	3.57	5.95	7.14	3.57	2.38	84
BAA81680.1	5.88	2.35	11.76	9.41	5.88	4.71	3.53	3.53	7.06	5.88	1.18	2.35	8.24	2.35	3.53	3.53	5.88	7.06	3.53	2.35	85
BAA81679.1	5.95	2.38	11.9	9.52	5.95	4.76	3.57	2.38	5.95	7.14	1.19	2.38	8.33	2.38	3.57	3.57	5.95	7.14	3.57	2.38	84
BAA81678.1	5.95	2.38	11.9	9.52	5.95	4.76	3.57	2.38	5.95	7.14	1.19	2.38	8.33	2.38	3.57	3.57	5.95	7.14	3.57	2.38	84
BAA81677.1	5.95	2.38	11.9	9.52	5.95	4.76	3.57	2.38	5.95	7.14	1.19	2.38	8.33	2.38	3.57	3.57	5.95	7.14	3.57	2.38	84
BAA81676.1	5.95	2.38	11.9	9.52	5.95	4.76	3.57	2.38	5.95	7.14	1.19	2.38	8.33	2.38	3.57	3.57	5.95	7.14	3.57	2.38	84
BAA81675.1	5.95	2.38	11.9	9.52	5.95	4.76	3.57	2.38	5.95	7.14	1.19	2.38	8.33	2.38	3.57	3.57	5.95	7.14	3.57	2.38	84
BAA81674.1	6.74	2.25	12.36	8.99	5.62	5.62	3.37	2.25	6.74	6.74	1.12	2.25	7.87	2.25	3.37	3.37	5.62	7.87	3.37	2.25	89
BAA81673.1	6.74	2.25	12.36	8.99	5.62	5.62	3.37	2.25	6.74	6.74	1.12	2.25	7.87	2.25	3.37	3.37	5.62	7.87	3.37	2.25	89
BAA81672.1	6.59	2.2	12.09	8.79	5.49	5.49	3.3	2.2	6.59	6.59	1.1	2.2	7.69	2.2	5.49	3.3	5.49	7.69	3.3	2.2	91
BAA81671.1	6.59	2.2	12.09	8.79	5.49	5.49	3.3	2.2	6.59	6.59	1.1	2.2	7.69	2.2	5.49	3.3	5.49	7.69	3.3	2.2	91
BAA81670.1	6.67	2.22	12.22	8.89	5.56	5.56	3.33	2.22	6.67	6.67	1.11	2.22	7.78	2.22	4.44	3.33	5.56	7.78	3.33	2.22	90
**Avg. %**	5.78	1.45	7.12	5.71	6.59	5.46	4.65	4.97	4.91	6.72	1.88	5.56	7.38	2.76	6.79	5.53	4.9	6.55	1.85	3.43	400.9

### Prediction of secondary structure

Predicting the secondary structure of proteins is critical to understanding protein folding in three dimensions. The secondary structure is predicted using the primary protein sequence [42]. Using SOPMA, the predicted secondary structure of protein sequences revealed the predominance of random coils with more than 40% except for a few sequences such as *Capsicum annuum*, *Solanum melongena*, *Solanum lycopersicum*, *Oryza meridionalis*, *Oryza rufipogon*, *Oryza glaberrima*, and *Oryza barthii*, which had extended arms in the majority. The alpha helix and beta turn found the highest repeats in *Populus deltoides* and *Oryza sativa*, as given in Table 5.

**Table 5:** Secondary structure prediction of plant catalases using SOPMA

Organism	Accession number	Alpha helix	Beta turn	Random coil	Extended strand
*Vitis vinifera*	AAL83720	27.03% (133)	7.7% (38)	48.78% (240)	16.46% (81)
*Vigna radiata*	ADZ455551	27.44% (135)	7.93% (39)	49.39% (243)	15.24% (75)
*Populus deltoides*	CAI439481	29.88% (147)	7.32% (36)	48.37% (238)	14.43% (71)
*Ziziphus jujuba*	AET975641	28.46% (140)	7.52% (37)	49.19% (242)	14.84% (73)
*Prunus persica*	CAD429091	27.85% (137)	7.32% (36)	48.98% (241)	15.85% (78)
*Phyllanthus emblica*	ATO983111	17.96% (29)	12.35% (21)	40.49% (69)	30% (51)
*Nicotiana plumbaginifolia*	CAA854261	27.03% (133)	6.91% (34)	50.81% (250)	15.24% (75)
*Bruguiera gymnorhiza*	ADC956291	28.86% (142)	7.93% (39)	47.15% (232)	16.06% (79)
*Arabidopsis thaliana*	CAA177731	27.64% (136)	7.93% (39)	48.78% (240)	15.65% (77)
*Raphanus sativus*	AAF717421	26.42% (130)	7.93% (39)	50.81% (250)	14.84% (73)
*Brassica juncea*	AAD179341	28.25% (139)	7.52% (37)	48.58% (239)	15.65% (77)
*Arabis alpina*	KFK301471	28.66% (141)	7.93% (39)	48.37% (238)	15.04% (74)
*Musa acuminata*	SIW589631	20.93% (54)	10.47% (27)	47.29% (122)	21.32% (55)
*Solanum tuberosum*	AAR140522	27.16% (129)	8% (38)	50.11% (238)	14.74% (70)
*Saccharum*	AIU994821	25.20% (124)	7.72% (38)	49.8% (245)	17.28% (85)
*Saccharum spontaneum*	AIU994861	25.81% (127)	7.72% (38)	49.59% (244)	16.87% (83)
*Saccharum arundinaceum*	AIU994841	28.05% (138)	7.72% (38)	48.17% (237)	16.06% (79)
*Oryza sativa*	BAA342041	27.44% (135)	8.13% (40)	47.76% (235)	16.67% (82)
*Triticum aestivum*	BAA130681	28.25% (139)	7.52% (37)	47.36% (233)	16.87% (83)
*Festuca arundinacea*	CAG239201	26.63% (131)	7.93% (39)	49.39% (243)	16.06% (79)
*Capsicum annuum*	AAF347181	29.07% (143)	6.91% (34)	14.02% (69)	50% (246)
*Solanum melongena*	CAA506441	26.83% (132)	6.71% (33)	16.06% (79)	50.41% (248)
*Solanum lycopersicum*	AAA341451	28.86% (142)	8.54% (42)	15.65% (77)	46.95% (231)
*Oryza meridionalis*	BAA816791	25% (144)	7.64% (44)	16.49% (95)	50.87% (293)
*Oryza rufipogon*	BAA816741	34.83% (31)	8.99% (8)	14.61% (13)	41.57% (37)
*Oryza glaberrima*	BAA816811	26.19% (22)	8.33% (7)	16.67% (14)	48.81% (41)
*Oryza barthii*	BAA816801	27.06% (23)	7.06% (6)	16.47% (14)	49.41% (42)

### Comparative homology modeling and its functional analysis

**Fig. 3:**
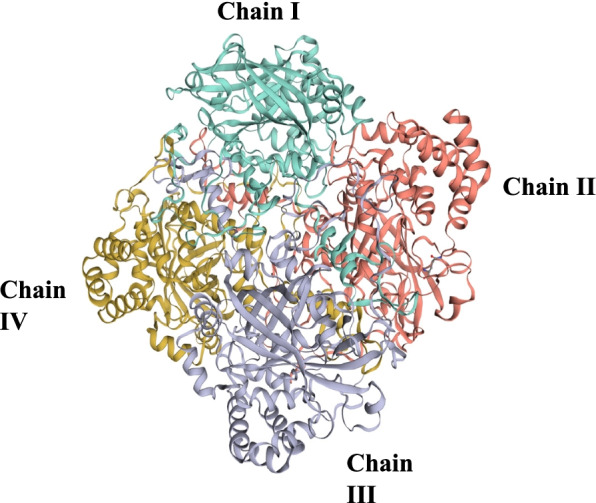
Predicted protein model of catalase enzyme of *Arabidopsis thaliana* showing distinct four homo-tetrameric chains

**Fig. 4:**
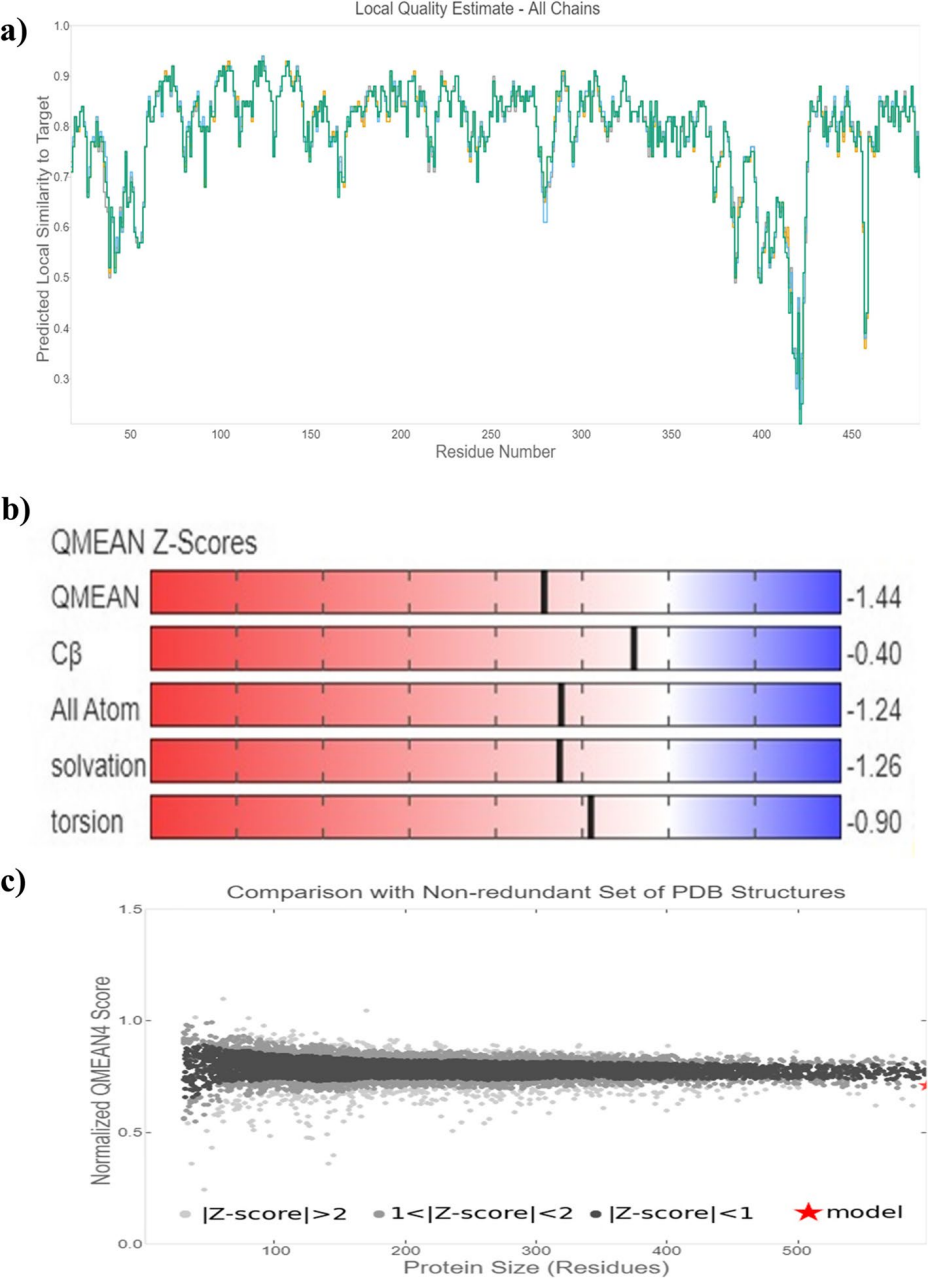
Predicted protein model quality estimation by SWISS-MODEL

**Fig. 5:**
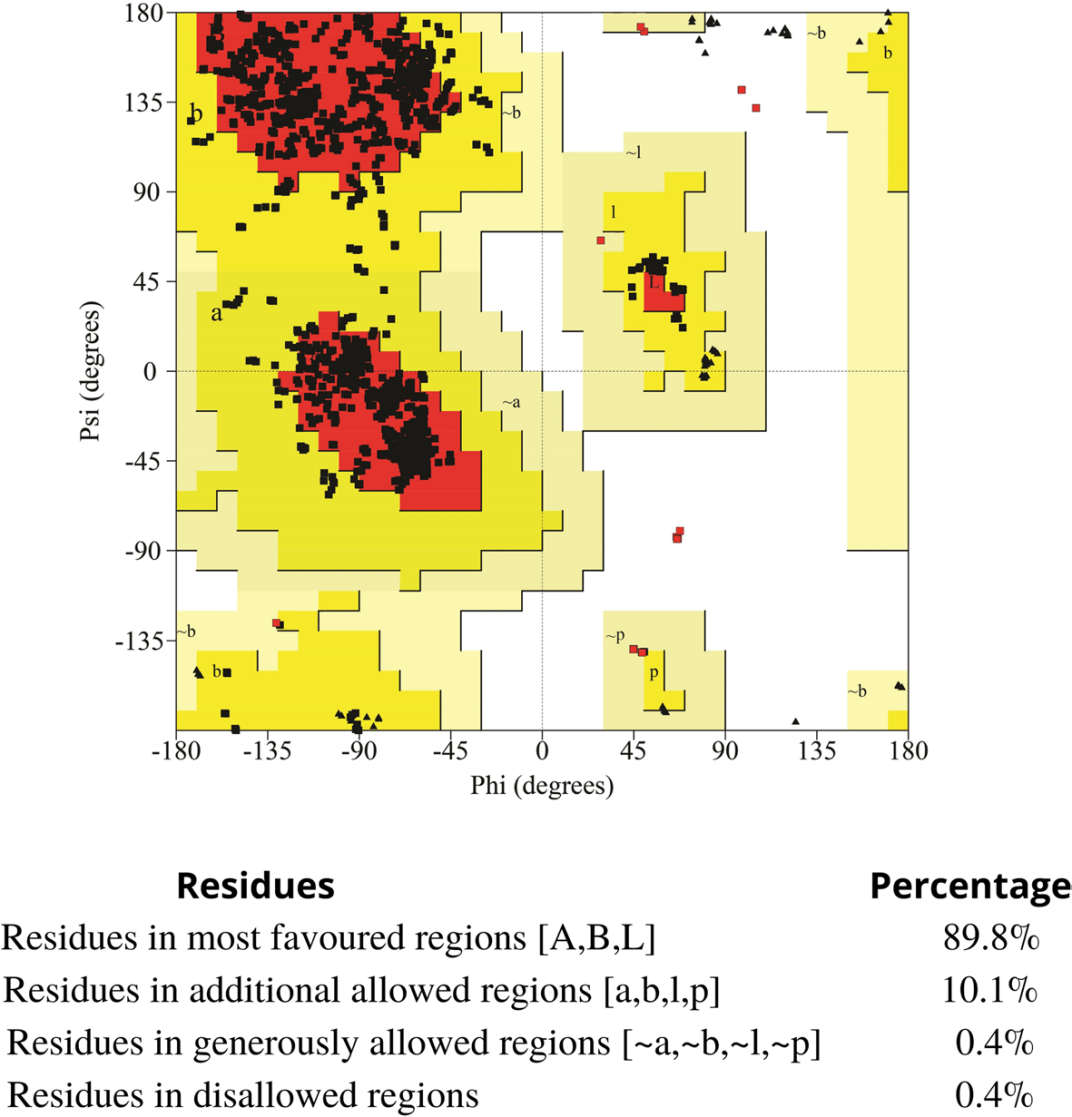
Ramachandran plot of predicted CAT model from *Arabidopsis thaliana* generated from PROCHECK. Residues in most favored regions (A, B, L)—89.8%. Residues in additional allowed regions (a, b, l, p)—10.1%. Residues in generously allowed regions (~a, ~b, ~l, ~p)—0.4%. Residues in disallowed regions—0.4%

To predict the 3D structure, a well-known template sequence is required, similar to the query sequence. A single organism from each cluster was selected, as shown in Table 6, and homology modeling of the 3D protein structure was carried out, where *Arabidopsis thaliana* was found as the query sequence to have the highest sequence identity and the GMQE score. The 3D structure was built by SWISS-MODEL using template 4qol.1.A *Bacillus pumilus* catalase by extrapolating experimental data from an evolutionarily related protein structure that serves as a template in Fig. 3, and the quality estimation of the predicted model is shown in Fig. 4a. The template’s sequence identity was 53.8% compared to the query sequence, the QMEAN score was −1.44, the GMQE value at 0.81 values, and the predicted model’s oligo state was homotetramer with 1.65 A resolution [43]. As part of the evaluation and validation process, the predicted protein model of the query sequence (in. PDB format) was uploaded to many servers. The Ramachandran plot analysis showed that 89.8% resided in the most favored (red) regions, while 10.1% fell into the additional allowed (brown) regions and 0.4% in the generously allowed regions, validating the quality of the modeled structure given in Fig. 5.

**Table 6:** Characterization of selected organism modeling from each cluster evaluated by SWISS-MODEL

Organism	Template	Residues	GMQE	Sequence identity (%)
*Vitis vinifera*	4qol.1.A	14-488	0.81	50.84
*Arabidopsis thaliana*	4qol.1.A	17-488	0.81	53.83
*Saccharum spontaneum*	4qol.1.A	14-490	0.81	51.99
*Triticum aestivum*	4qol.1.A	18-487	0.80	53.32
*Solanum tuberosum*	4qol.1.A	17-488	0.80	50.32
*Oryza sativa japonica*	4qol.1.A	14-489	0.81	46.86

**Fig. 6:**
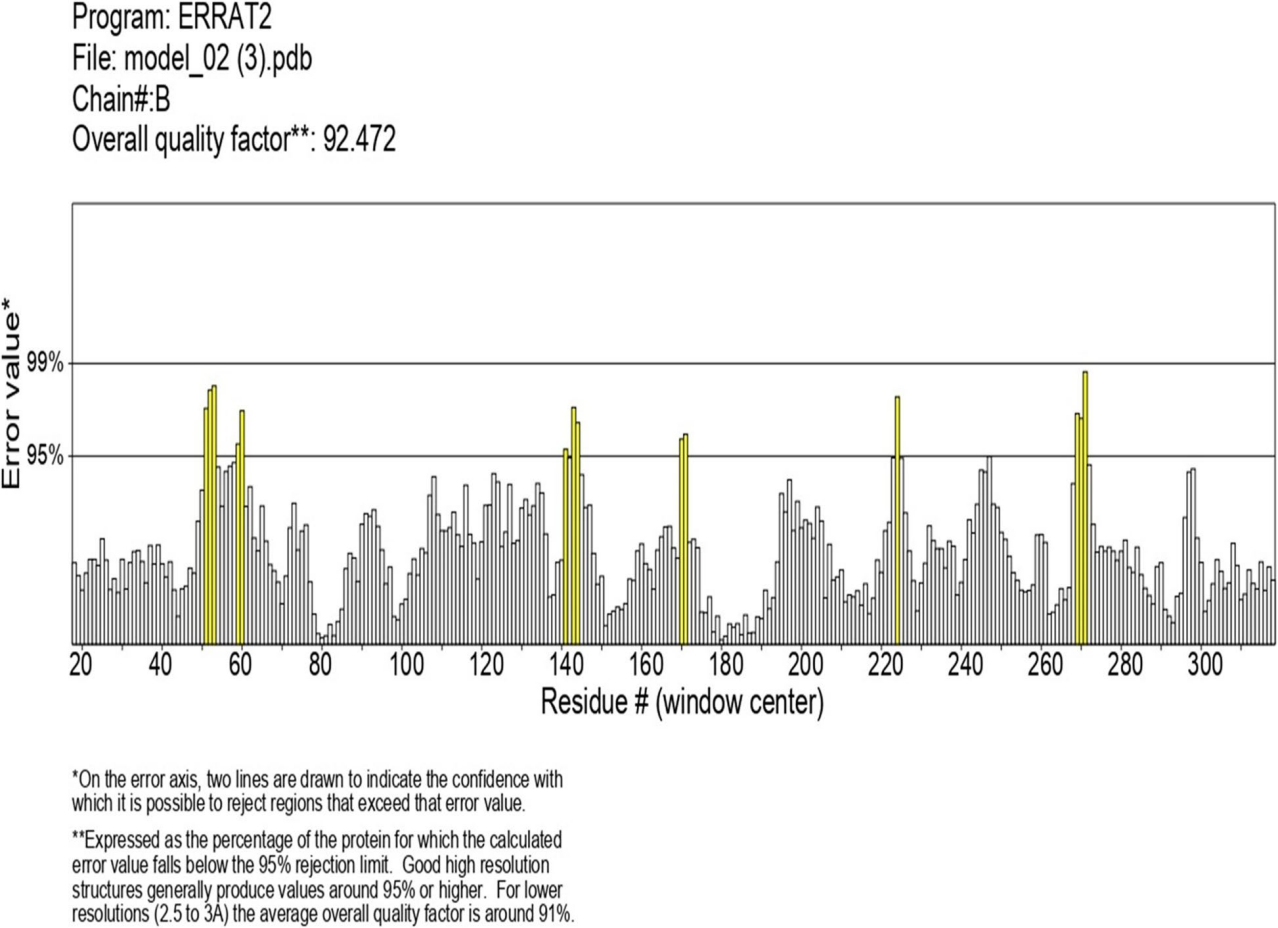
ERRAT plot of *Arabidopsis thaliana* catalase model with overall quality factor 92.47

**Fig. 7:**
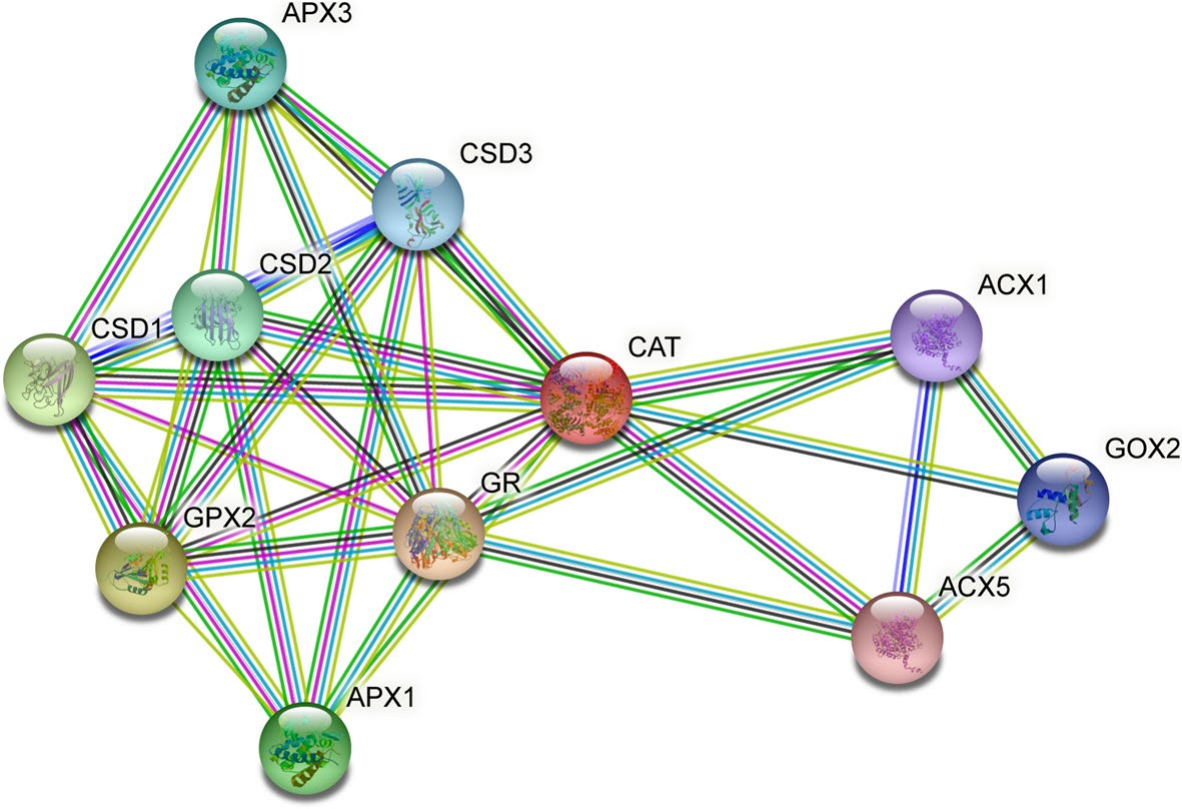
Map of the protein-protein interaction of *Arabidopsis thaliana* catalase protein

**Fig. 8:**
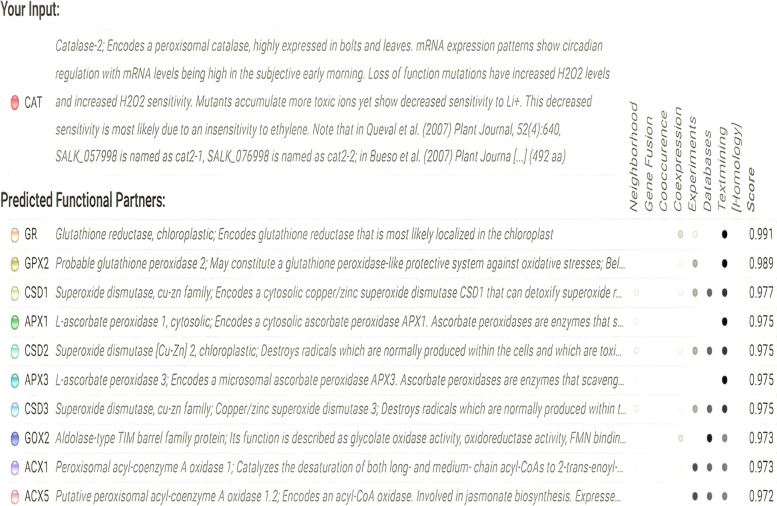
Predicted interacting protein partners of the query sequence from STRING server

The overall G factor of dihedral angles and covalent forces was −0.16, higher than the allowable threshold of −0.5. A high G factor indicates that a stereochemical characteristic correlates with a high probability of conformation [44, 45]. The predicted model was submitted to the SAVES server. ERRAT plots were used to examine the protein model’s atom distribution with one another and to make decisions regarding the model’s reliability when evaluating the amino acid environment. The overall quality factor of ERRAT was 92.5, indicating a slightly negligible value of the individual residues (Fig. 6). The Verify3D suggested that the CAT model has at least 80% of amino acids with a score > = 0.2 in the 3D/1D profile, while the average residue was around 70.2%, suggesting the compatibility of the predicted model with its amino acid residues [46]. The QMEAN Z-score in Fig. 4b and c was −1.4, which was in the expected range of 0.0 to −2.0, representing a well-defined structure [47]. The cellular machinery is built on a foundation of proteins and their functional relationships. It is necessary to consider a network of webs between organisms to understand biological phenomena. The STRING analysis revealed ten predicted interacting partners of query CAT protein from the organism *Arabidopsis thaliana* (accession number CAA45564.1), which encodes peroxisomal catalase and revealed glutathione reductase as the closest interacting protein with the shortest distance. On the contrary, ACX5 (putative peroxisomal acyl-coenzyme A oxidase) remained distant from the query protein (Figs. 7 and 8) [48].

## Discussion

Computational approaches have established themselves as a valuable complement to our understanding of the protein universe and its properties. In silico analysis is one of the most helpful tools that contributes significantly to computational biology for exploring the structural and functional properties of the protein. Hence, the study was conducted to explore the structural and functional properties of catalase enzymes from plants using different bioinformatics tools such as ProtParam, MEGA-X, SOPMA, SWISS-MODEL, and SAVES server. The Expasy tool revealed several physiochemical characteristics of the retrieved catalase sequences, each representing its unique behavior. The pH at which a protein does not have a net electrical charge and is considered neutral is known as its isoelectric or isoionic point [49]. In the development of buffer systems for purification and isoelectric focus, the prediction of pI is critical. The study suggested that the theoretical pI value of most plant catalases is acidic ranging from 5 to 7, but *Capsicum annuum* has an alkaline pI value of 7.11. The instability index of protein catalases ranged from 28.94 to 44.90, except for a few species of catalases having an index of more than 40 with accession number CAD42908, CAD42909 (*Prunus persica*), AAD17934, AAD17935, AAD17938 (*Brassica juncea*), KFK30147 (*Arabis alpina*), CAA85424 (*Nicotiana plumbaginifolia*), BAF91369, AAF34718 (*Capsicum annuum*), BAA81682, BAA81681 (*Oryza glaberrima*), and BAA81680 (*Oryza barthii*). The aliphatic index refers to the percentage of a protein’s total volume occupied by its hydrophobic aliphatic side chains. The heat stability of a protein depends on its aliphatic index. A higher aliphatic index means that proteins are better able to withstand high temperatures [50]. Catalases with an aliphatic index ranging from 65.66 to 75.55 have substantial amounts of hydrophobic amino acids and are very thermally stable. The hydrophilic nature of the plant catalases was observed with the GRAVY score. The GRAVY negative score indicates that the protein could be globular (hydrophilic) rather than membranous (hydrophobic). This information could aid in the identification of these proteins [51]. The phylogenetic tree analysis was constructed using the maximum likelihood method to show evolutionary relationships among plant catalases. The distribution of *Oryza sativa* in different clusters C, D, and F revealed its genetic diversity and similarity with *Festuca arundinacea* and *Saccharum spontaneum*. Using a Pfam database search and NCBI/CDD-BLAST, the proteins were categorized into specific families based on the presence of a specific domain of their sequences. The NCBI BLAST designated the PLN02609 superfamily for catalase proteins with conserved domains. Overlapping annotations on the same protein sequences are generated by a superfamily, which is a collection of conserved models that have evolutionary domains. Protein secondary structure prediction from sequences is regarded as a link between the prediction of primary and tertiary structures [52]. Based on catalase secondary structure prediction, it was revealed the predominance of random coils followed by alpha helix in most of the catalases [3], which is highly similar to the results of CAT1 genes of PgCAT1, *Soldanella alpina*, and *Gossypium hirsutum* [7]. Random coils are irregular secondary arrangements found in the N and C terminal arms and loops of the protein structure occur because of electrostatic repulsion and steric hindrance of bulky adjacent residues such as isoleucine or charged residues such as glutamic acid or aspartic acid. In a random coil state, the average conformation of each amino acid residue is independent of the conformations of all residues other than those immediately proximal in the primary structure [53]. The amino acid composition of plant catalases revealed the highest proline content, which could explain the predominant coiled structural content. Proline has the unique ability to cause coiling by disrupting secondary conformations by causing kinks in polypeptide chains [54]. In silico prediction of a 3D model of a protein is a difficult element of correlating data received from NMR or crystallography-based approaches [48]. The query sequence (CAA45564) was blasted against PDB to find the best template. The highest sequence identity of 53.8% with negative QMEAN value and GMQE score suggested the template selection 4qol.1.A of *Bacillus pumilus* catalase. The validation of the predicted structure was performed by computational tools where 89.8% favored region of Ramachandran plot implied good quality of the model. The SAVES server tools ERRAT, Verify3D, and QMEAN Z-scores suggested a well-defined protein structure. The functional relationships of our query sequence revealed the glutathione reductase as the closest interacting protein with the shortest distance, which may be associated with the overlapping of its functional roles in the metabolic pathway [55].

## Conclusion

In silico analysis of plant catalase protein provides insight into the numerous catalytic sites, allowing for possible manipulation of desirable qualities relevant to various sectors. Phylogenetic analysis revealed the similarity of various plant catalases, elucidating how species evolve genetically. Scientists can use phylogenetics to determine the genetic link between a modern organism and its ancestral origin and anticipate future genetic divergence. Numerous conserved amino acid residues among distinct clusters may allow for developing particular probes or markers that reflect source species from a specific taxon. Secondary structure analysis confirmed the predominance of a random coil followed by an alpha helix, an extended strand, and a beta turn. Plant catalases had the highest proline content in their amino acid composition, which could explain their coiled structural content. Proline has the unique ability to cause coiling in polypeptide chains by disrupting secondary conformations. The predicted 3D CAT model from *Arabidopsis thaliana* was a homotetramer, thermostable protein with 59-KDa weight, and its structural validation was confirmed by PROCHECK, ERRAT, Verify3D, and Ramachandran plot. In silico protein structure analysis is an extremely valuable technique for exploring protein structure-function relationships when crystal structures are unavailable. It can also help predict ligand-receptor interactions, enzyme-substrate interactions, mutagenesis experiments, SAR data, and loop structure prediction. While these studies build a robust foundation for wet-lab experimentation, they also provide a strong framework for looking at novel sources utilizing metagenomics approaches and directed evolution to incorporate desired functional qualities.

## Data Availability

Not applicable
